# Usefulness of decision tree analysis of MRI features for diagnosis of placenta accreta spectrum in cases with placenta previa

**DOI:** 10.1007/s11604-024-01684-3

**Published:** 2024-11-06

**Authors:** Yasuhiro Tanaka, Hirofumi Ando, Tsutomu Miyamoto, Yusuke Yokokawa, Motoki Ono, Ryoichi Asaka, Hisanori Kobara, Chiho Fuseya, Norihiko Kikuchi, Ayumi Ohya, Yasunari Fujinaga, Tanri Shiozawa

**Affiliations:** 1 Department of Obstetrics and Gynecology, Shinshu University School of Medicine, 3-1-1 Asahi, Matsumoto, Nagano, 390-8621 Japan; 2 Department of Radiology, Shinshu University School of Medicine, 3-1-1 Asahi, Matsumoto, Nagano, 390-8621 Japan

**Keywords:** Placenta previa, Placenta accreta spectrum, Magnetic resonance imaging, Decision tree analysis, Preoperative diagnosis

## Abstract

**Purpose:**

Placenta previa complicated by placenta accrete spectrum (PAS) is a life-threatening obstetrical condition; therefore, preoperative diagnosis of PAS is important to determine adequate management. Although several MRI features that suggest PAS has been reported, the diagnostic importance, as well as optimal use of each feature has not been fully evaluated.

**Materials and methods:**

The occurrence of 11 PAS-related MRI features was investigated in MR images of 145 patients with placenta previa. The correlation between each MRI feature and pathological diagnosis of PAS was evaluated using univariate analysis. A decision tree model was constructed according to a random forest machine learning model of variable selection.

**Results:**

Eight MRI features showed a significant correlation with PAS in univariate analysis. Among these features, placental/uterine bulge and myometrial thinning showed high odds ratios: 138.2 (95% CI: 12.7–1425.6) and 66.0 (95% CI: 18.01–237.1), respectively. A decision tree was constructed based on five selected MRI features: myometrial thinning, placental bulge, serosal hypervascularity, placental ischemic infarction/recess, and intraplacental T2 dark bands. The decision tree predicted the presence of PAS in the randomly assigned validation cohort with significance (*p* < 0.001). The sensitivity and the specificity of the decision tree for detecting PAS were 90.0% (95%CI: 53.2–98.9) and 95.5% (95%CI: 89.9–96.8), respectively.

**Conclusion:**

Among PAS-related MRI features, placental/uterine bulge and myometrial thinning showed high diagnostic values. In addition, the present decision tree model was shown to be effective in predicting the presence of PAS in cases with placenta previa.

## Introduction

Placenta accreta spectrum (PAS) is an obstetrical complication in which abnormal trophoblasts invade into, or even through the myometrium and cause massive postpartum hemorrhage [1, 2]. The most important risk factor for the development of PAS is placenta previa after a prior cesarean delivery. The frequency of PAS rises with an increasing number of previous cesarean sections. A large prospective study reported that zero, one, two, three, four, five, or a greater number of previous cesarean sections were comorbid with PAS in 3, 11, 40, 61, and 67 percent of placenta previa cases, respectively [3]. As cases of cesarean delivery have increased in recent decades, the prevalence of PAS has markedly increased [4, 5]. In a 2019 systematic review that included 7001 cases of PAS among nearly 5.8 million births, the overall pooled prevalence of PAS was 0.17 percent (range: 0.01 to 1.1 percent) [6].

Methods for the prediction of PAS in patients with placenta previa cases have long been desired because management can change markedly in PAS cases. In the case of placenta previa without PAS, bleeding after detaching the placenta can often be conservatively controlled [7, 8]. However, when placenta previa is complicated by PAS, any attempt to detach the placenta must be performed with special care because of the risk of massive fatal hemorrhage [9]. Depending on the severity of the condition, a cesarean hysterectomy or expectant management with or without endovascular intervention can be selected in cases where a preoperative diagnosis has been made [10, 11]. Therefore, the preoperative diagnosis of placenta previa with or without PAS has a critical influence on the clinician’s decision-making when planning the surgical strategy.

Several reports have attempted to preoperatively detect PAS using MRI. In recent years, there has been a shift from focusing on individual MRI findings to the accumulation of multiple findings as represented by the Society of Abdominal Radiology and European Society of Urogenital Radiology (SAR-ESUR) consensus statement 2020 [12–20]. It is useful to list several MRI features for the preoperative diagnosis of PAS, which may contribute to making the diagnosis more precise. However, it is rare for a single case to have all the features, and in the majority of cases, only a few of the 11 MRI features listed in the SAR-ESUR statement are present. Therefore, it is important to elucidate the degree of importance of each feature regarding its diagnostic value.

Decision tree analysis is a common data mining technique that is often employed to formulate prediction algorithms for specific variables. The method divides the population into branch-like sections, forming an inverse tree consisting of a root node, internal nodes, and leaf nodes. The algorithm is nonparametric, does not require complex parametric structures, and can efficiently handle complex datasets [21]. By employing a decision tree model, MRI features can be weighted according to their relative importance. Consequently, prediction of the presence of PAS can be achieved. However, to date, there has been no report on the construction of a decision tree model using MRI features to predict the presence of PAS in placenta previa cases.

In this study, we aimed to evaluate MRI features to predict placenta previa with PAS comprehensively and weigh each feature to develop a decision tree that is useful to clinicians when making decisions on operative procedures.

## Materials and methods

### Patients

One hundred and forty five pregnant women with placenta previa diagnosed using MRI who visited Shinshu University Hospital between 2008 and 2022 were retrospectively reviewed. The patient data including the age at diagnosis, number of previous cesarean sections, main placental position, and post-operative diagnosis were collected from medical records.

### MRI and image analysis

MRI was performed at a median gestational age of 32 weeks (interquartile range, IQR: 30–34). We analyzed the features in sagittal, axial, and coronal T2-weighted images (T2WIs) and diffusion-weighted images. MR imaging was performed using a 1.5 T MR imaging system (Magnetom AVANTO or Magnetom AVANTO Fit, Siemens Healthineers AG, Germany). We obtained T2WIs using a half-Fourier acquisition single-shot turbo spin-echo (HASTE) sequence and true fast imaging with steady-state precession (TrueFISP) sequence. All MR images were obtained at a section thickness of 5 mm with a 1 mm intersection gap. Sequence parameters of each image were as follows: axial and sagittal, coronal T2-weighted HASTE: TR/TE, 500 or 1200/79–80 ms; field of view, 280 × 380–420 × 420 mm, axial and sagittal, coronal T2-weighted TrueFISP: TR/TE, 4.87–5.05/2.13–2.44 ms; field of view. 337.5 × 360–420 × 420 mm, diffusion-weighted echo-planar imaging with b values of 0 and 1000: TR/TE, 4300 or 6100 ms; field of view, 320 × 250–450 × 351.563 mm. An experienced radiologist (A.O.) and an obstetrician–gynecologist (H.K.), who were blinded to patient identifiers, clinical information, and the pathological diagnosis, assessed the MR images of the lesions focused on the features listed in the following section. Each feature was assessed by five-point Likert scale scores (1: definitely negative, 2: probably negative, 3: neutral, 4: probably positive, 5: definitely positive). The scores 1 to 3 were interpreted as negative and 4 to 5 as positive for the presence of the feature. Disagreement regarding the features between the two readers was resolved by discussion and consensus.

### Definitions of the signs of placenta accreta spectrum in MR imaging

**Fig. 1:**
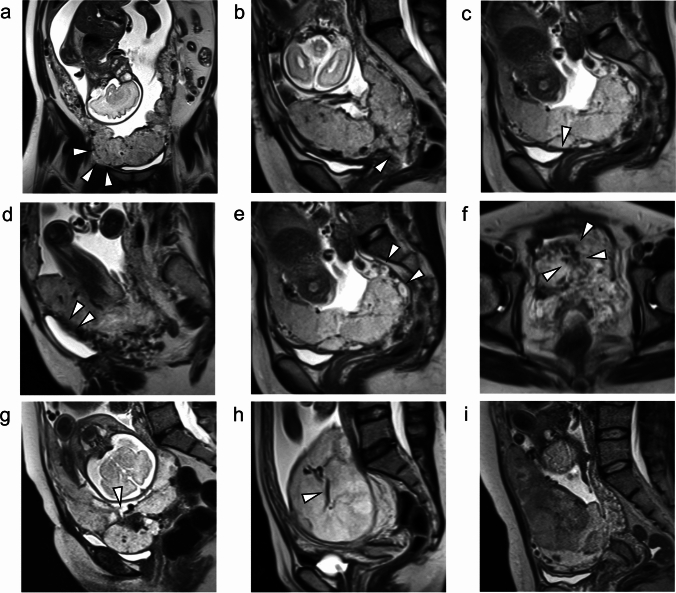
Representative MRI (T2WI) features of PAS. **a** Placental/Uterine bulging (arrowheads). **b** Placental protrusion into the internal os (arrowhead). **c** Myometrial thinning or focal disruption (arrowhead). **d** Loss ofT2 hypointense interface (arrowheads). **e** Myometrial hypervascularity (arrowheads). **f** Serosal hypervascularity (arrowheads). **g** Placental ischemic infarction/recess and intraplacental T2 dark bands (arrowhead). **h** Abnormal intraplacental vascularity (arrowhead). **i** Placental heterogeneity. All images shown were acquired with single-shot turbo spin-echo (HASTE) sequence

We developed a list of MRI features to detect PAS in placenta previa cases with a modification of previous reports [13–18] and the SAR-ESUR consensus statement [12] (Table 1). The modification included the use of two subcategories: myometrial hypervascularity and serosal hypervascularity, to replace the category “abnormal vascularization of the placental bed”, which is used in the SAR-ESUR consensus statement. The scanned images were re-evaluated to determine the existence of the following 11 MRI features in 3 categories: 1. Gross morphological signs, including placental/uterine bulging (Fig. 1a), placental protrusion into the internal ostium of uterus (internal os) (Fig. 1b), and percretism signs (not shown). 2. Interface signs, including myometrial thinning or focal disruption (Fig. 1c), loss of T2 hypointense interface (Fig. 1d), myometrial hypervascularity (Fig. 1e), and serosal hypervascularity (Fig. 1f). 3. Tissue architecture signs, including intraplacental T2 dark bands (Fig. 1g), placental ischemic infarction/recess (Fig. 1g), abnormal intraplacental vascularity (Fig. 1h), and placental heterogeneity (Fig. 1i).

**Table 1:** Definition of MRI features for prediction of PAS with previa

1.	Gross morphological signs
	A) Placental/Uterine bulging*: loss of normal “pear shape” of the uterus, with enlargement of the fundus and wider appearance of the body than caudal segments
	B) Placental protrusion into the internal os: extension and projection of the placenta into the internal uterine os
	C) Percretism signs: direct invasion of the placenta into adjacent organs (bladder or rectum), which results in exophytic placental tissue protruding through the uterine wall and extending beyond it. The involved bladder may show a “tenting” shape
2.	Interface signs
	A) Myometrial thinning or focal disruption*: focal defect of the hypointense uteroplacental interface with myometrial thinning (≤ 1 mm) or indistinctness of myometrial delineation on T2-weighted images
	B) Loss of T2 hypointense interface*: loss of visualization of the T2 hypointense inner myometrial layer underneath the placenta, which represents normal sub-placental vascularity
	C) Myometrial hypervascularity: increased and dilated vasculature in the myometrium of the placental bed
	D) Serosal hypervascularity: increased and dilated vasculature on the serosal surface of the uterus
3.	Tissue architecture signs
	A) Intraplacental T2 dark bands*: band-shaped T2 hypointense lines in the placenta (width ≥ 2 cm)
	B) Placental ischemic infarction/recess*: recess of the placental surface (fetal side or myometrium) accompanying a T2 dark band. This essentially represents infarction related to volume loss
	C) Abnormal intraplacental vascularity*: enlarged and tortuous vessels represented by flow void signals with a diameter > 6 mm within the placenta on T2-weighted images
	D) Placental heterogeneity*: heterogeneous intensity within the placenta due to hemorrhage or vascular lacunae

*Asterisked MRI features are adapted from the SAR-ESUR consensus statement

*PAS* placenta accreta spectrum

### Statistical analysis

The correlation of each MRI feature and pathological diagnosis of PAS was analyzed by Fisher’s exact test using the open-source statistical computing environment R (version 4.2.1; R Foundation for Statistical Computing). Haldane’s correction, which adds 0.5 to each cell of the 2 by 2 table, was employed in the case that one of the cells was zero [22]. Decision tree analysis was conducted following previous reports [23, 24]. The relative importance of MRI features was evaluated according to the rank of mean decrease in Gini impurity using random forest modeling built by the R package “randomForest”. Consecutively, a decision tree model constituted by the selected MRI features was constructed using JMP Pro 16 (SAS Institute Inc., Cary, NC, USA). In short, patients were randomly assigned to the derivation or validation cohort at a ratio of 2:1. Using the derivation cohort, a decision tree model was built using 5 MRI features selected by random forest analysis. The sensitivity, the specificity, and the area under the curve (AUC) of the receiver operating characteristic (ROC) curve of the decision tree were calculated using data from the validation cohort.

## Results

### Patients

The characteristics of the patients are summarized in Table 2. The ages of the patients and gestational ages at the time of MRI were not significantly different between the PAS and non-PAS groups. The number of previous cesarean sections was significantly higher in cases with PAS (*p* < 0.001). The anterior position of the main part of the placenta was more frequently observed in cases of PAS (*p* < 0.001). Twenty-three cases underwent cesarean hysterectomy and were diagnosed with PAS pathologically. Two patients who underwent cesarean section without hysterectomy were surgically diagnosed with PAS because the placenta strongly attached to the uterus, and defect of the myometrial layer after detaching of the placenta was noted.

**Table 2:** Patient characteristics

	All cases (*n* = 145)	Non-PAS cases (*n* = 120)	PAS cases (*n* = 25)	*p*-value
Age (median, IQR)	35 (31–37)	35 (31–37)	34 (33–38)	0.787
Number or previous cesarean sections				< 0.001
0	93 (64.1%)	92 (76.7%)	1 (4%)	
1	30 (20.7%)	17 (14.2%)	13 (52%)	
2	20 (13.8%)	10 (8.3%)	10 (40%)	
3	2 (1.4%)	1 (0.8%)	1 (4%)	
Gestational age at MRI scan (weeks) (median, IQR)	32 (30–34)	32 (30–34)	31 (29–33)	0.139
Main placental position				< 0.001
Anterior	65 (44.8%)	46 (38.3%)	19 (76%)	
Posterior	80 (55.2%)	74 (61.7%)	6 (24%)	
Post-operative diagnosis				
Placenta previa without PAS	120 (82.8%)	120 (100%)	0	
Placenta accreta	16 (11.0%)	0	16 (64%)	
Placenta increta	4 (2.8%)	0	4 (16%)	
Placenta percreta	5 (3.4%)	0	5 (20%)	

*IQR* inter quartile range, *PAS* placenta accreta spectrum

### MRI features

**Fig. 2:**
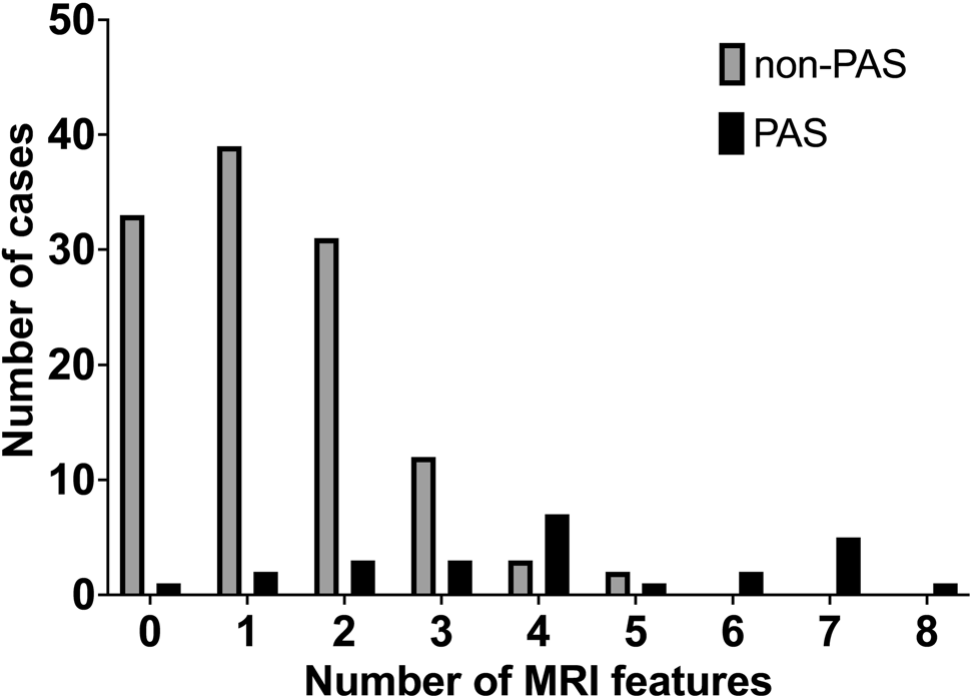
Number of MRI features observed in individual cases of PAS and non-PAS

The frequency of occurrence of each MRI feature ranged from 0.7 to 45.5%. The relatively frequently observed MRI features were: myometrial hypervascularity (45.5%), intraplacental T2 dark bands (26.9%), heterogenous placenta (24.1%), and myometrial thinning (23.4%) (Table 3). The number of MRI features found in cases of placenta previa with PAS was significantly higher than in those without PAS (mean ± SD 4.2 ± 2.2 and 1.3 ± 1.2, *p* < 0.001) (Fig. 2).

**Table 3:** The frequency of MRI features in patients

	All cases (*n* = 145)	Non-PAS cases (*n* = 120)	PAS cases (*n* = 25)
Placental/Uterine bulge	10 (6.9%)	0 (0%)	10 (40.0%)
Percretism sign	1 (0.7%)	0 (0%)	1 (4.0%)
Placental protrusion	11 (7.6%)	5 (4.2%)	6 (24.0%)
Myometrial thinning	34 (23.4%)	12 (10.0%)	22 (88.0%)
Loss of T2 hypointense interface	8 (5.5%)	1 (0.8%)	7 (28.0%)
Myometrial hypervascularity	66 (45.5%)	58 (48.3%)	8 (32.0%)
Serosal hypervascularity	23 (15.9%)	10 (8.3%)	13 (52.0%)
Placental ischemic infarction/ recess	26 (17.9%)	13 (10.8%)	13 (52.0%)
Intraplacental T2 dark bands	39 (26.9%)	23 (19.2%)	16 (64.0%)
Abnormal intraplacental vascularity	13 (9.0%)	8 (6.7%)	5 (20.0%)
Heterogeneous placenta	35 (24.1%)	29 (24.2%)	6 (24.0%)

*PAS* placenta accreta spectrum

### Correlation of MRI features and presence of PAS

**Fig. 3:**
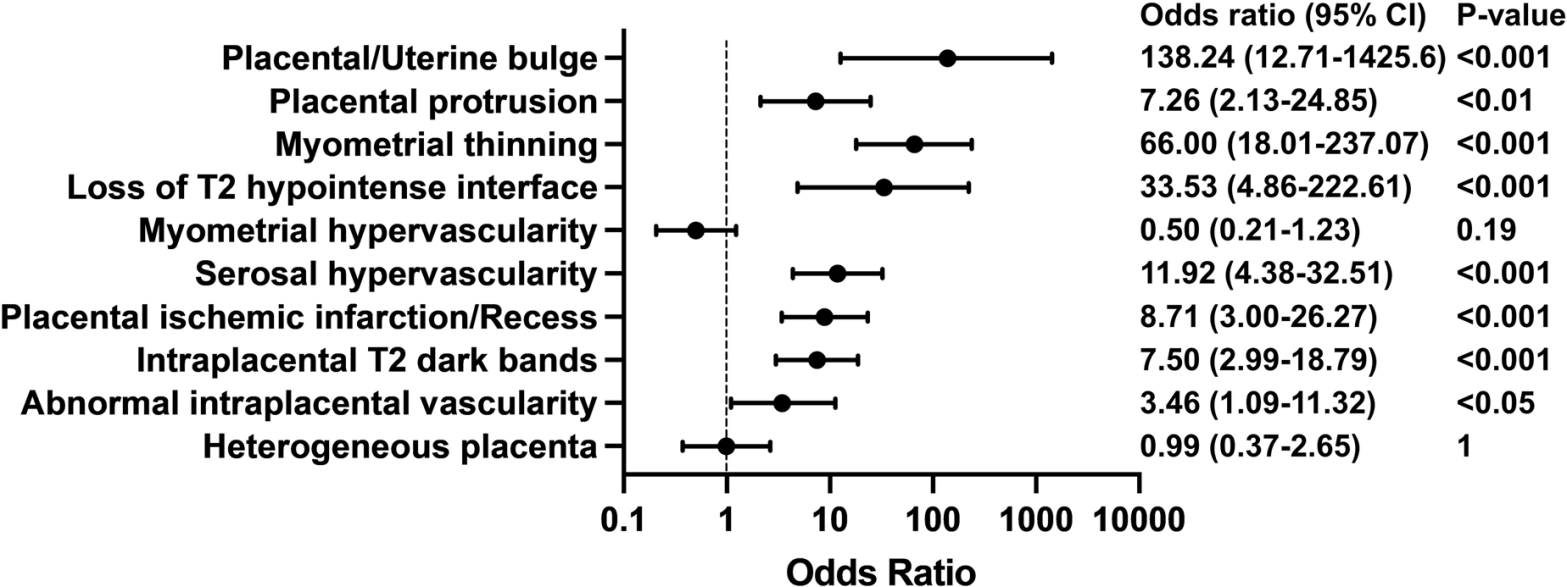
Forest plot of the univariate analysis for each MRI feature. Mean odds ratios and 95% confidence intervals of MRI features for post-operative histopathological diagnosis of PAS and p values from Fisher’s exact test for each MRI finding are shown

We conducted a univariate analysis for 10 MRI features, excluding the percretism sign, because this feature was observed in only 1 case. The analysis revealed significant correlations with PAS for 8 MRI features, whereas myometrial hypervascularity and heterogenous placenta failed to show significance. Of those features that showed significant differences, placental/uterine bulge, myometrial thinning, and loss of the T2 hypointense interface showed prominently high odds ratios: 138.24 (95% CI: 12.71–1425.6), 66.00 (95% CI: 18.01–237.07), and 33.53 (95% CI: 4.86–222.61), respectively. Serosal hypervascularity ranked after these three (OR 11.92, 95% CI: 4.38–32.51). The odds ratios and the p values of Fisher’s exact test are graphically summarized in Fig. 3. The other statistical values (sensitivity, specificity, positive/negative predictive values, and positive/negative likelihood ratios) of each MRI feature are shown in Table 4. Of note is that the single MRI features with a high odds ratio indicate high specificity, but not high sensitivity enough to make diagnosis of PAS.

**Table 4:** Results of contingency table analysis

	Odds ratio (95% CI)	Sensitivity (95% CI)	Specificity (95% CI)
Placental/Uterine bulge	138.2 (12.71–1425)	0.396 (0.283–0.414)	0.995 (0.97–1)
Placental protrusion	7.263 (2.125–24.85)	0.240 (0.129–0.342)	0.958 (0.935–0.98)
Myometrial thinning	66.00 (18.01–237.1)	0.880 (0.731–0.956)	0.900 (0.869–0.916)
Loss of T2 hypointense interface	33.53 (4.859–222.6)	0.261 (0.154–0.296)	0.990 (0.964–0.998)
Myometrial hypervascularity	0.503 (0.206–1.232)	0.320 (0.178–0.498)	0.517 (0.487–0.554)
Serosal hypervascularity	11.92 (4.382–32.51)	0.520 (0.364–0.655)	0.917 (0.884–0.945)
Placental ischemic infarction/Recess	8.917 (3.422–23.31)	0.520 (0.361–0.664)	0.892 (0.858–0.922)
Intraplacental T2 dark bands	7.498 (2.989–18.79)	0.640 (0.468–0.784)	0.808 (0.773–0.838)
Abnormal intraplacental vascularity	3.500 (1.092–11.32)	0.200 (0.096–0.325)	0.933 (0.912–0.959)
Heterogeneous placenta	0.991 (0.373–2.653)	0.240 (0.110–0.409)	0.758 (0.733–0.793)

	Positive predictive value (95% CI)	Negative predictive value (95% CI)	Positive likelihood ratio (95% CI)	Negative likelihood ratio (95% CI)
Placental/Uterine bulge	0.959 (0.680–0.995)	0.879 (0.857–0.883)	83.92 (0.392–835.1)	0.607 (0.586–0.739)
Placental protrusion	0.545 (0.292–0.777)	0.858 (0.837–0.877)	5.760 (1.980–16.70)	0.793 (0.672–0.932)
Myometrial thinning	0.647 (0.537–0.703)	0.973 (0.939–0.990)	8.800 (5.577–11.36)	0.133 (0.048–0.310)
Loss of T2 hypointense interface	0.857 (0.505–0.974)	0.848 (0.826–0.856)	25.04 (4.265–156.9)	0.747 (0.705–0.878)
Myometrial hypervascularity	0.121 (0.068–0.189)	0.785 (0.740–0.841)	0.662 (0.348–1.116)	1.316 (0.906–1.687)
Serosal hypervascularity	0.565 (0.396–0.712)	0.902 (0.870–0.929)	6.240 (3.149–11.869)	0.524 (0.365–0.719)
Placental ischemic infarction/Recess	0.500 (0.347–0.639)	0.899 (0.866–0.929)	4.800 (2.548–8.489)	0.538 (0.364–0.745)
Intraplacental T2 dark bands	0.410 (0.300–0.502)	0.915 (0.875–0.949)	3.339 (2.058–4.846)	0.445 (0.258–0.688)
Abnormal intraplacental vascularity	0.385 (0.184–0.624)	0.848 (0.829–0.872)	3.000 (1.083–7.974)	0.857 (0.704–0.992)
Heterogeneous placenta	0.171 (0.085–0.292)	0.827 (0.800–0.866)	0.993 (0.448–1.978)	1.002 (0.745–1.201)

### Decision tree analysis

**Fig. 4:**
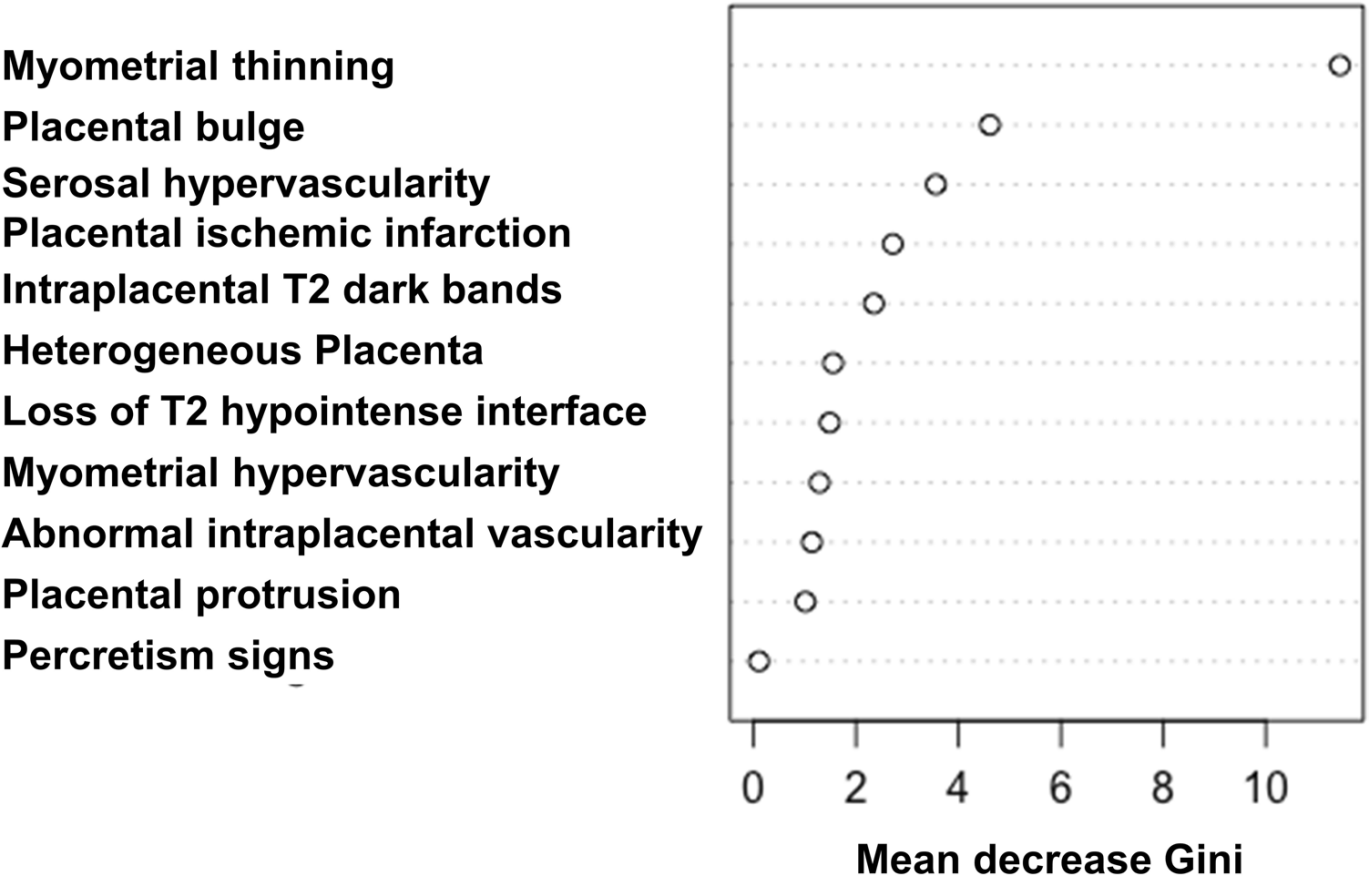
The mean decrease in impurity (Gini) of each MRI feature

**Fig. 5:**
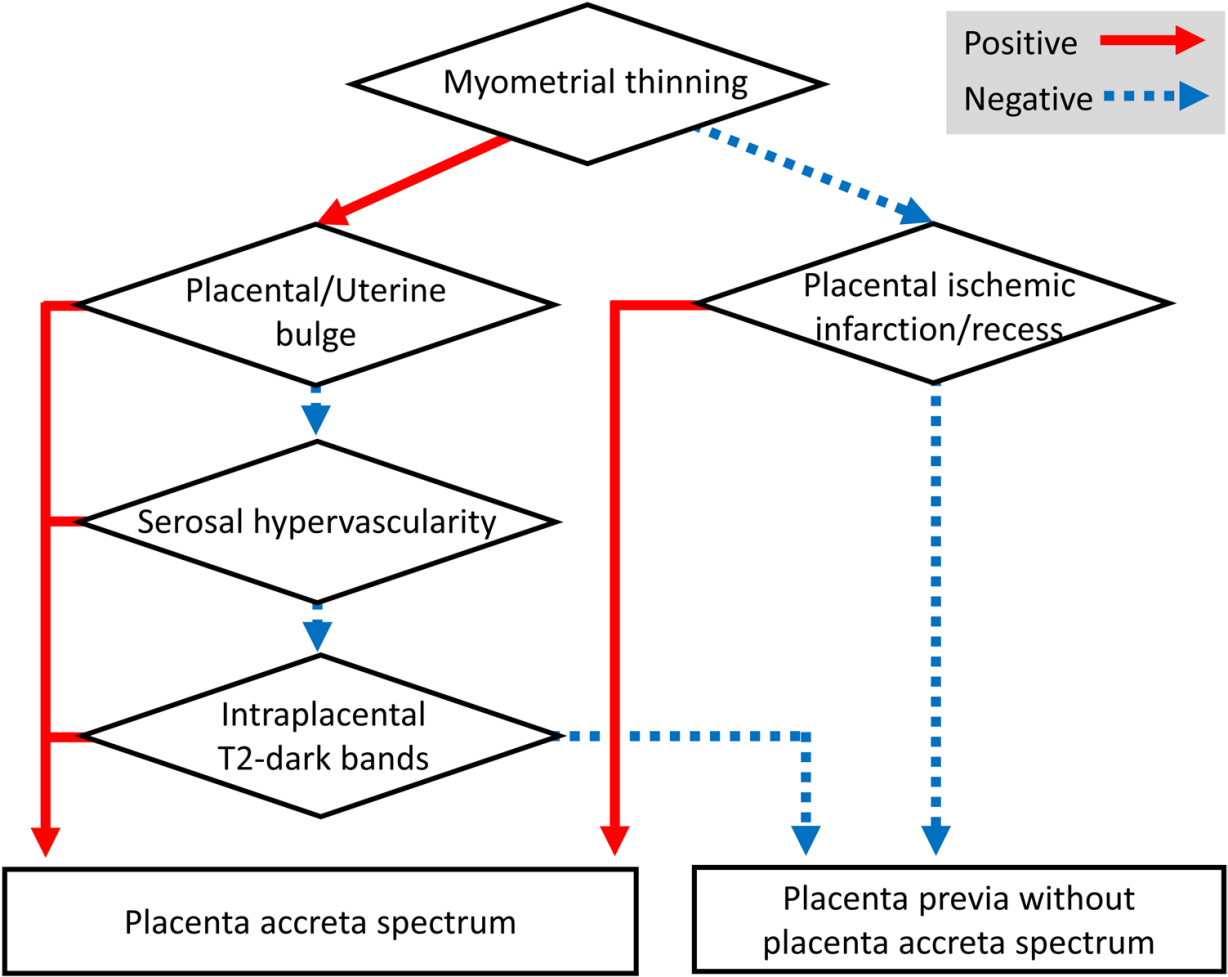
The decision tree for interpreting multiple MRI features for preoperative diagnosis of PAS

**Fig. 6:**
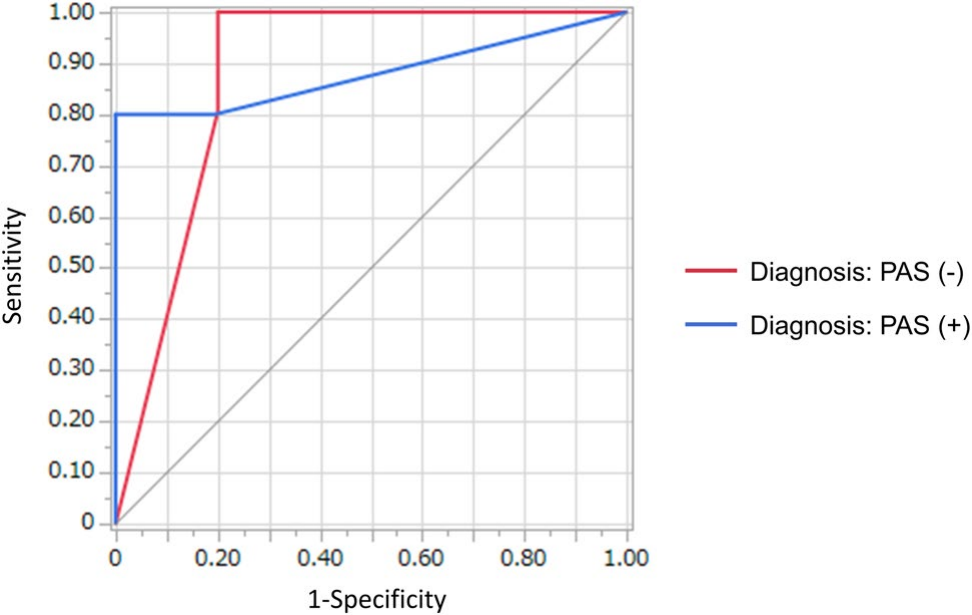
ROC curves of the decision tree applied to data from the validation cohort

Next, we constructed a decision tree model for the prenatal diagnosis of PAS. First, the mean decrease in impurity (Gini) of each feature was calculated by the random forest model (Fig. 4). According to the rank of this analysis, myometrial thinning, placental bulge, serosal hypervascularity, placental ischemic infarction/recess, and intraplacental T2 dark bands were selected. Next, the patients were randomly assigned to the derivation or validation cohort. The decision tree was developed as shown in Fig. 5. The decision tree predicted the presence of PAS in the validation cohort with significance (*p* < 0.001, Fisher’s exact test). The sensitivity, the specificity, the positive predictive value, and the negative predictive value of the decision tree for the detection of PAS were: 90.0% (95%CI: 53.2–98.9), 95.5% (95%CI: 89.9–96.8), 75.0% (95%CI: 44.4–82.4), and 98.4% (95%CI: 92.7–99.8), respectively. AUC of the ROC curve was 0.881 (Fig. 6).

## Discussion

In the present study, we evaluated eleven MRI features for the preoperative diagnosis of PAS, including signs that were previously reported and newly proposed criteria; myometrial, and serosal hypervascularity. Accordingly, the significance of serosal hypervascularity together with the other signs previously reported was validated by univariate analysis, and their diagnostic potentials were confirmed. In addition, this study also proposes a decision tree to integrate MRI features, by which PAS can be predicted with high specificity. To the best of our knowledge, this is the first report to propose the use of a decision tree model for preoperative diagnosis of placenta previa with PAS.

In this study, we used newly proposed criteria: myometrial and serosal hypervascularity, as a replacement for the MRI feature “abnormal vascularization of the placental bed”, which was proposed in the SAR-ESUR consensus statement [12]. The criterion “abnormal vascularization of the placental bed” is defined in the SAR-ESUR consensus statement as follows: “Prominent vessels in the placental bed with disruption of the uteroplacental interface. They may extend to the underlying myometrium to a variable degree, reaching up to the uterine serosa; and may be accompanied by extensive neovascularization around the bladder, uterus, and vagina”. According to this description, abnormal vascularization may exist in a variety of tissues, including the muscular or serosal layer of the uterus and adjacent organs. It also may cause confusion as the description includes features used in other criteria, such as myometric disruption and invasion of other organs. As our best efforts to avoid mixed criteria, we divided abnormal vascularization of the placental bed into two categories: “myometrial hypervascularity” and “serosal hypervascularity”. In addition, cases with myometrial disruption by vascularity were categorized in “myometrial thinning/disruption” and those with obvious invasion into other organs were included in “percretism signs”. The univariate analysis revealed a significant correlation between placenta previa with PAS and eight MRI features including serosal hypervascularity. However, myometrial hypervascularity and heterogeneous placenta failed to show a significant correlation with PAS. This finding suggests the importance of distinguishing the layer of the uterus which shows abnormal vascularity.

We developed a decision tree model to integrate the MRI features, which comprised five MRI features: myometrial thinning, placental/uterine bulge, serosal hypervascularity, intraplacental T2 dark bands, and placental ischemic infarction/recess. The sensitivity and the specificity of the decision tree in the validation cohort were 90.0% (95% CI: 53.2–98.9) and 95.5% (95% CI: 89.9–96.8), respectively. Maurea et al. retrospectively evaluated 61 placenta previa patients who underwent pathological diagnosis and reported that the sensitivity and the specificity of MRI to detect PAS were respectively 100 and 53%, when the diagnosis was made in cases with at least one of the MRI features. When the diagnosis was made in cases with at least two of the MRI features observed, the sensitivity and the specificity were respectively 92 and 92%. Although comparison of diagnostic accuracy between reports is difficult because the MRI features included in the reports were different and a limited number of subjects were evaluated in each study, the decision tree that the present study proposed showed favorable accuracy.

In this report, the variable selection for the decision tree excluded three (placental protrusion, loss of T2 hypointense interface, and abnormal intraplacental vascularity) of the MRI features. Variable selection was a necessary step to prevent overfitting of the decision tree to the derivation cohort and improve the predictive accuracy for unknown cases, but it was not for determining the individual diagnostic value of these excluded MRI features. Because the selection of MRI features was determined by the decrease in impurity (Gini), which was calculated using random forest analysis, the relative importance would be influenced by the combinations of MRI features found in each case in the cohort. In other words, if a feature can be substituted by different features, its relative importance in selecting the feature may be reduced. For example, the MRI feature “loss of T2 hypointense interface” showed a significantly high odds ratio in univariate analysis but failed to be selected in the features of the decision tree. We consider it may be because “loss of T2 hypointense interface” frequently observed in combination with myometrial thinning and/or placental/uterine bulge. On the other hand, the MRI feature “placental ischemic infarction/recess” was selected for the decision tree. This feature showed a relatively low odds ratio in the univariate analysis and was also categorized as “uncertain” and not as “recommended” in the consensus statement of SAR-ESUR [12]. However, the consensus statement collected experts’ opinions about each feature and classified its category. Placental ischemic infarction/recess is thought to reflect reduced remodeling of the spiral arteries in the PAS and consequent abnormal blood flow between the uterus and placenta and within the placenta [25–27]. Therefore, it is considered an important finding closely related to the pathophysiology of the PAS. Therefore, we consider “placental ischemic infarction/recess” important in combination with other features. Of note, among the 145 cases analyzed in this study, 6 cases (5 cases of heterogenous placenta and 1 case of placental protrusion) were found to have only the three excluded features without the five features included in the decision tree, and the final diagnosis was non-PAS in all cases. This suggests that PAS is unlikely to be present when only these features are present.

Although we conducted the random assignment of the derivation and the validation cohort, this study has limitations from the retrospective nature. Also, the detection of MRI features has a subjective nature and interobserver variability.

In conclusion, the decision tree we propose has the potential to integrate MRI features and predict PAS with high specificity. Further study is needed to evaluate the usefulness of this decision tree for the clinical diagnosis of placenta previa with PAS and comprehensively evaluate the MRI features obtained.
